# Downward Rotation of the Capsulopalpebral Fascia, Orbital Septum, and Orbital Fat Complex: A New Technique for Lower Eyelid Rejuvenation

**DOI:** 10.1097/GOX.0000000000002335

**Published:** 2019-08-08

**Authors:** Zhenying Huang, Yongkuk Lee, Gen Yan, Kai Wang

**Affiliations:** From the * Department of Cosmetic Surgery, Congro Marie Women and Children’s Hospital, Tianjin Province, China; † Department of LH Plastic Surgery Clinic, Seoul, Korea; ‡ Department of Radiology, The Second Affiliated Hospital of Xiamen Medical College, China; § Department of Plastic Surgery, Department of International Medical Center, Henan Provincial People’s Hospital, Zhengzhou University, China.

## Abstract

Filling of the orbitomalar sulcus through fat conservation and relocation is important for the lower eyelid rejuvenation procedure and has been reported on extensively. This study aimed to introduce a new technique—downward rotation of the capsulopalpebral fascia, septum, and fat complex in lower eyelid blepharoplasty to correct orbitomalar sulcus depression.

**Methods:**

Eighty-six patients who underwent transcutaneous lower eyelid blepharoplasty for cosmetic purposes from March 2015 to March 2016 were included in this study. The results were evaluated based on pre- and postoperative photographs, surgical records, and questionnaires.

**Results:**

The patients had no permanent or major complications. There were no fat hernia recurrences, diplopia, fat granulomas, or soft tissue irregularities. Approximately 98% of the patients were satisfied with the outcome.

**Conclusions:**

This technique more completely fills the orbitomalar sulcus and reinforces the anterior wall of the lower lid septum with capsulopalpebral fascia by rotating the orbital fat downward with the septum and capsulopalpebral fascia. Thus, it lowers the recurrence rate of the lower lid fat hernia and does not require fat removal. In particular, it has a distinct advantage in terms of the correction of the orbitomalar sulcus depression in reoperation cases, especially in patients who undergo fat removal or those with excessive fat removal.

## INTRODUCTION

A single convex line without signs of bony landmarks presents a youthful contour appearance of the eyelid-cheek complex.^1^ Gradually, midcheek aging is characterized by progressive anatomic landmark changes, such as eye bags and lower lid orbitomalar sulcus depression (tear trough and lid-cheek junction),^2–4^ which present a tired appearance. The correction of those landmarks is very important in improving facial rejuvenation.^5,6^

In the history of lower blepharoplasty technique advances, there have been many surgical techniques designed to improve the most effective aesthetic results and to prevent postoperative complications.^7^ Surgical fat removal with lower lid skin excision was first performed in the early evolution of prominent lower eyelid fat management.^8–11^ Further, Loeb has popularized the fat sliding technique to achieve a more natural and comprehensive rejuvenation of the lower eyelid region.^1,12–14^ De la Plaza and Arroyo have been interested in fat preservation and tightening of the orbital septum techniques,^15–17^ because they described their capsulopalpebral fascia (CPF) repair technique for lower eyelid herniation in 1988. Although several surgical techniques are available, the correction of orbital malar sulcus depression is still a challenge.

In this article, the downward rotation technique of the capsulopalpebral fascia, orbital septum, and orbital fat complex to orbitomalar sulcus depression can address a change of eyelid contour and create a more youthful and pleasing appearance. We describe this new surgical technique for lower eyelid blepharoplasty to preserve the lower eyelid fat and to more fully correct orbitomalar sulcus depression. In particular, this technique decreases the recurrence rate of palpebral bags.

## METHODS

### Patient Selection

Patients who underwent lower eyelid blepharoplasty using the transcutaneous approach from March 2015 to March 2016 were included in this study. This study was approved by ethics committee of Congro Marie Women and Children’s Hospital, and we applied informed consent to all the patients. The patients did not have systemic health, psychiatric, or ophthalmologic problems. All patients had positive lower lid tilting, without scleral show, and showed 6 mm in the snap-back test, along with good elasticity in the lower eyelid pinch test.

The indications for surgery were as follows: (1) those requiring skin resection due to sagged or residual skin; (2) those with clear orbital malar sulcus depression deformity; and (3) those with clear fat hernia. Of these, the first case is an indispensable indication for external incision surgery. If there is no skin sagging or residual skin, the transconjuctival route should be considered. If there is a lower lid malposition, the corresponding surgery, such as canthopexy or canthoplasty, should be performed simultaneously.

### Operation Method

The operation was performed while the patients were in a sitting position. The position of the orbitomalar sulcus depression, the borderline below the pretarsal roll, and the position and size of fat bulging were marked. Some patients were placed under monitored anesthetic care or anesthesia, but most of them were administered local anesthesia. After local anesthesia administration, 4- to 5-mm subcutaneous tissues were dissected using fine-curved scissors through a subciliary incision. Next, the septum was exposed by incising the orbicularis oculi muscle downward. With the skin-muscle flap held, dissection was performed along the surface of the septum by the spreading action of the mosquito forceps just above the arcus marginalis. Below the arcus marginalis, hemostasis and blunt dissection were performed along with the suborbicularis oculi muscle layer. In the middle portion, dissection was performed about 6–8 mm below the arcus margin, bypassing the arcus marginalis. In the medial part, the medial nasal attachments of the orbicularis oculi were released, and a pocket was created up to the medial part 4–5 mm of the orbital rim. In the lateral part, the lateral orbicularis ligaments were released, and dissection was performed along with suborbicularis oculi layer. Depending on the degree of sagging of the malar soft tissue, the suborbicularis pocket was enlarged or became small. At this time, supraperiosteum or suborbicularis tissues should be as thick as possible to facilitate the downward rotation tie-over of the capsulopalpebral fascia, septum, and fat complex. The dissection area varied depending on the degree of skin sagging. However, if dissection was performed excessively, the risk of innervation to the orbicularis nerve was increased. Therefore, in general, it was performed within 5 mm of the orbital rim in the medial portion and within 6 mm below the arcus marginalis in the medial portion. In the lateral portion, inferior and lateral dissection was more extensively performed to suspend the orbicularis oculi muscle in many cases. Since there may be long-term sensory abnormalities after surgery, it should be performed within 1.0 cm of the lateral portion of the orbital rim. Once dissection around the orbital rim was complete, the capsulopalpebral fascia dissection was begun.

**Fig. 1.**
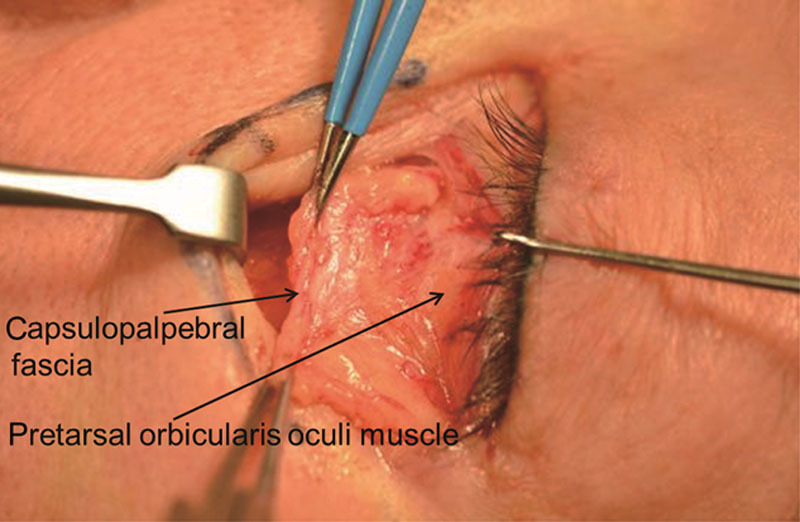
Retracted capsulopalpebral fascia and pretarsal orbicularis oculi muscle.

**Fig. 2.**
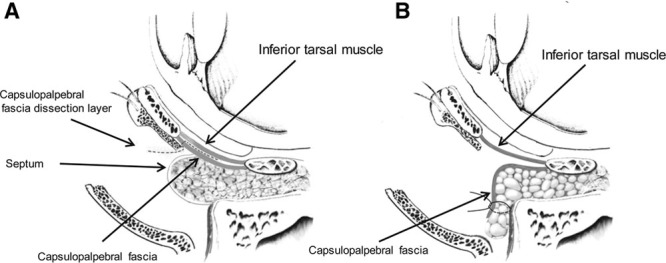
Capsulopalpebral fascia, septum, and fat complex is rotated downward and fixed to the lower orbital rim. A, capsulopalpebral fascisia predissection view; marked intended dissection layer; B, capsulopalpebral fascia, septum, and fat complex is rotated downward and fixed to the lower bital rim.

When pulling the pretarsal orbicularis oculi muscle and the capsulopalpebral fascia underneath it in opposite directions, a border between the capsulopalpebral fascia and the pretarsal orbicularis oculi muscle was shown in the middle (Fig. 1). At this state, careful dissection using the Bovie or scissors along the back of the capsulopalpebral fascia led to the capsulopalpebral fascia, septum, and fat complex (Fig. 2).

The capsulopalpebral fascia was dissected sufficiently beyond the arcus marginalis to cover the tear trough and lid-cheek junction depression deformity when the capsulopalpebral fascia, septum, and fat complex were rotated downward. In most cases, dissection was performed up to the fornix.

The capsulopalpebral fascia was pulled down, and if tension was present on the lower eyelid, it was released to eliminate the tension. In this case, the lateral and medial horns^3^ of the capsulopalpebral fascia were dissected to allow more freedom of the capsulopalpebral fascia, thereby making the capsulopalpebral fascia, septum, and fat complex flat to easily reach beyond the arcus marginalis. In the medial and lateral portions of the orbitomalar sulcus, release of the septum at the site connected with both horns of the capsulopalpebral fascia released the septum and fat complex, resulting in a free-tension septum and fat complex covering the medial and lateral portions of the orbitomalar sulcus. Whether to cut the medial and lateral horns depended on whether the length of the capsulopalpebral fascia, septum, and fat complex flap could sufficiently cover the orbitomalar sulcus during surgery.

**Fig. 3.**
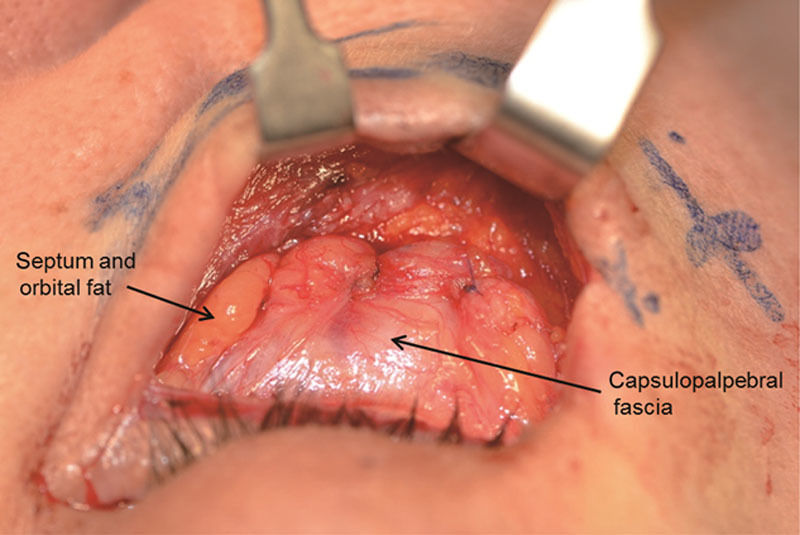
Intraoperative image after the downward rotation of the capsulopalpebral fascia, septum, and fat complex.

After dissection of the capsulopalpebral fascia, the capsulopalpebral fascia, septum, and fat complex flap was pulled to the extent of 5–6 mm below the arcus marginalis to release tension, and the fan-shaped vascularized capsulopalpebral fascia, septum, and fat complex flap was closed using interrupted 6-0 Vicryl sutures by 4–6 points on the tissues above the periosteum (Fig. 3).

The medial and lateral portions of the orbitomalar sulcus, which were not reached due to the narrow capsulopalpebral fascia, were covered with the released septum and fat complex if necessary (if depression was present) and sutured to the tissues above the periosteum using 6-0 Vicryl. Gentle pressure was placed on the upper eyelid over the globe to examine for fat bulging. If bulging is present, plication of the capsulopalpebral fascia and septum through a horizontal suture reinforces the anterior wall of the septum, which helps in preventing fat hernia recurrence. Next, skin-muscle flap redraping was performed, and the residual skin and muscles resulting from the incision were wound inward with the patient’s eyes closed. After marking the skin to be incised, the skin was conservatively removed. In patients who complained of oversized pretarsal roll before surgery, an appropriate amount of pretarsal muscle was excised. Meticulous hemostasis was attained with bipolar cautery. Next, as a routine, the lateral portion of the orbicularis oculi muscle was sutured to the lateral periosteal orbital rim with a superior and slight lateral vector. Horizontal mattress suturing was performed with 3-0 Vicryl in 1-4 places (degree depended on skin sagging before surgery and dissection of skin-muscle flap). After confirming the absence of lower lid malposition, the skin carefully underwent interrupted suturing.

### Postoperative Care

After the operation, an ice pack was applied on the medial canthal to the lateral canthal along with the orbital rim for 10 minutes. Then, the patient was transferred to the recovery room; with the head elevated at 30 degrees, another ice pack was applied on the same area. After finding no hemorrhage and discomfort in visual acuity or the eye, they were returned.

The patients were instructed to apply an ice pack once every hour within 24 hours after the operation and were administered antibiotics for 3 days after the surgery. They were followed up on the first and fourth day postoperatively, and incision care was performed. On the seventh day, the stitches were removed, and the operative site was checked on the 10th day. Thereafter, follow-up examinations were performed at 3 weeks, 3 months, 6 months, and 1 year, postoperatively.

## RESULTS

A total of 86 patients who underwent transcutaneous lower eyelid blepharoplasty from March 2015 to March 2016 were included in this study. Two patients were male, and 84 were female, with a mean age of 46 years (range 31–63 years). All patients were of Asian ethnicity. Follow-up was not carried out according to the plan due to the nature of the cosmetic surgery. Sixteen of the 86 patients did not return to the hospital 7 days after the operation, that is, after removing the stitches. Only 19 of all study patients were followed up for 3 or more months. The mean statistics of follow-up time in all 86 patients was 64.5 days.

After surgery, short-term chemosis happened in 6 patients, which lasted 2 months in 2 cases. Five patients showed mild lower lid retraction, but they recovered within 2 months and had no lower eyelid ectropion. There was no postoperative bleeding; however, 4 patients had mild conjunctival hematoma, but recovered within 3 weeks. None of the patients complained of dry eye syndrome or discomfort. Three patients who had a wide dissection area due to severe skin ptosis complained of sensory abnormalities (two: 6 months and one: 9 months), but the abnormalities were not severe. Two patients underwent reoperation due to skin ptosis, and 1 underwent reoperation due to pretarsal roll asymmetry. There were no fat hernia recurrences, diplopia, fat granulomas, or soft tissue irregularities. Approximately 98% of the patients were satisfied with the outcome.

## DISCUSSION

To achieve the effective youthful contour rejuvenation of the lower eyelid complex, the lower blepharoplasty has been referred to a more challenging procedure with improvement of the aesthetic outcomes and prevention of postoperative complications. We have described this new approach in the article (downward rotation technique of capsulopalpebral fascia, orbital septum, and orbital fat complex), which can address those specific problems during operation.

Traditional lower eyelid blepharoplasty focused on surgical excision of the skin, muscle, and fat pads to improve the palpebral bulge.^14,18–20^ However, this procedure often led to long-term problems, such as lower lid malposition, scleral show, rounded palpebral fissures, and degree of hollow lower lid area, and a sunken appearance of the globe.^20^

Subsequently, the approach to handling orbital fat pads has changed in cosmetic blepharoplasty. Fat repositioning technique with less aggressive fat resection was described by Loeb in 1981 and the transferring of orbital fat pads was advocated to fill the tear trough depression.^14^ According to the description of operation method, the orbital fat pedicle is freed using cutting cautery. The incomplete blood supply probably causes a combination of postsurgical fibrosis, liponecrosis, and lipogranuloma formation. Somehow, the ischemia and variable absorption can occur in the fat pedicle. Generally, another problem is the fat pedicle is not enough for transposition to the depression of lower lid orbitomalar sulcus.^21^

Recently, the management of fat pads in lower lid blepharoplasty has changed to the fatty tissue relocation and the mere alteration of the tensile integrity of the orbital septa.^22^ de la Plaza and Arroyo,^15^ Mendelson,^16^ and Camirand et al.^23^ described the approach of suturing the capsulopalpebral fascia to the arcus marginalis to relocate the herniated fat pads. Sensoz et al.^24^ reported the approach of suturing the septum to the orbital periosteum to ameliorate the fat pad protrusion.

However, it is not possible to reconstruct the periorbital region with herniated fatty tissue relocation, which should lead to the consideration of other fat-preserving techniques.^17^ With aging, the supporting structures of the globe demonstrate progressive distention, which contributes to a caudal displacement of the globe and fat protruding.^15,22^ Relocation of fat pads without recovering the caudal displacement of the globe increases the pressure of fat pads to the anterior wall, resulting in the recurrence of palpebral bulge, especially at the lateral region of the lower lid with weakened septa.

In 1982, Hawes and Dortzbach^25^ described the capsulopalpebral fascia in microscopical level. In 1983, they first described the capsulopalpebral fascia as a legitimate structure.^26^ Meanwhile, in 1994, Goldberg et al. demonstrated that the capsulopalpebral fascia can be outlined as a valid construct by ultrafine surface coil MRI.^27,28^

**Fig. 4.**
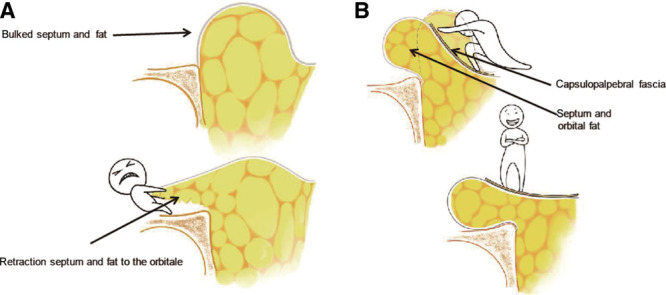
Diagram demonstrating the difference between the fat and septum transposition technique (A) and the capsulopalpebral fascia, septum, and fat complex downward rotation technique (B).

In contrast to others, the downward rotation technique of the capsulopalpebral fascia, orbital septum, and orbital fat complex is considered to have several advantages over other techniques (Fig. 4). First, this procedure does not require any incision to open the septum or manage the orbital fat pads. As described by Huang in 2000,^22^ limited surgical manipulation of the orbital septum and the orbital fat pads can minimize these undesirable consequences, such as increased risk of fat injury, degree of hollow lower lid area, or sunken appearance of the globe. Moreover, lid malposition resulting from scar contraction of the middle lamellar region, hemorrhage of the fat tissues, and fat granulomas resulting from lack of fat circulation are avoided. Second, the capsulopalpebral fascia is dissected as a flap to alter tensile integrity of the orbital septa, preventing the recurrence of lower lid palpebral bulge. Meanwhile, release of the capsulopalpebral fascia decreases the risk of lower lid malposition, scleral show, rounded palpebral fissures, and limitation of the upward mobility of the lower eyelids. Third, lengthening of the capsulopalpebral fascia helps the capsulopalpebral fascia, orbital septum, and orbital fat complex achieve tear trough and lid-cheek junction to fill the depression fully, resulting in a minor youthful convexity of the eyelid. But according to the reports of Plaza,^15^ Mendelson,^16^ and Sensoz et al.,^24^ this minor youthful convexity cannot be achieved by suturing the CPF to the arcus marginalis or the septum to the orbital periosteum. Finally, rotation of herniated orbicularis preseptal fat to the inferior position of the orbital rim reduces either the lower lid palpebral bulge or the pressure of fat pads to the anterior wall, resulting in a decreased risk of recurrence of palpebral bulge. In the long-term follow-up study of lower blepharoplasty with CPF by Parsa in 2008,^29^ there was 30.8% recurrence rate of palpebral bags in standard fat resection side, and 7.7% in CPF hernia repair side. But we have never observed any recurrence case yet, even we do not have as long as 10 years follow-up time. Our rotation method transfers all the pressure of fat pads to the anterior wall of the inferior position, meanwhile the septum of lower eyelid is gained in strength with CPF flap. Hence, we believe that the recurrence risk of palpebral bags is very low following the downward rotation technique of the CPF, orbital septum, and orbital fat complex.

## CONCLUSIONS

The capsulopalpebral fascia, septum, and fat complex downward rotation technique does not require the removal of the fat in the septum, and it fully corrects the orbitomalar sulcus depression while totally preserving the fat. It can partially fill the volume of the depressed orbitomalar sulcus in the reoperation of patients with excessively removed fat. It is a stable technique that can prevent fat hernia recurrence and the hollowed-out appearance of the orbit in the lower lid blepharoplasty.

**Fig. 5.**
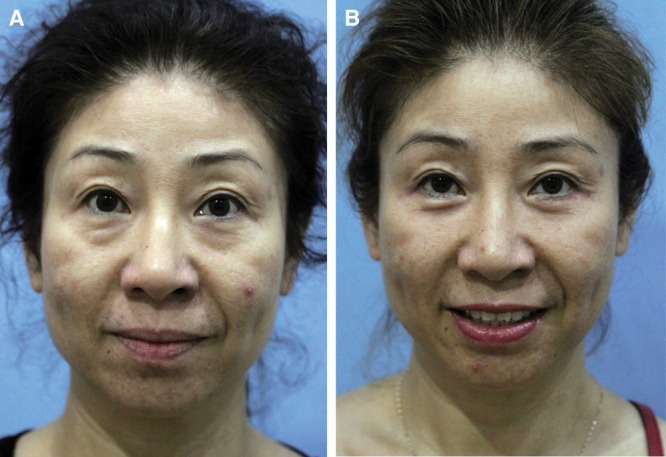
Preoperative image of a woman (A). Six months after lower blepharoplasty (B), there is an improvement in the orbitomalar sulcus.

**Fig. 6.**
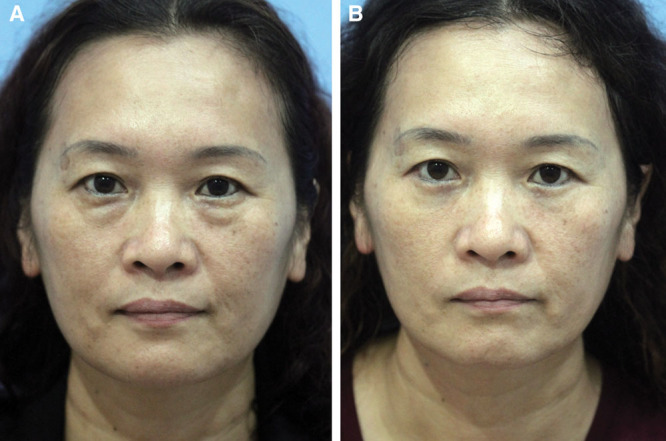
Preoperative image of a woman (A). One year postoperatively (B), there is an improvement in the orbitomalar sulcus

## ACKNOWLEDGMENTS

We are very grateful to all participants in this study. We also thank Mr. Hongsen Shao for helping us in our figure artwork.

Patients provided written consent for the use of their images.
